# Modification mechanism of calcium lignosulfonate on cementing cement

**DOI:** 10.1038/s41598-024-58077-9

**Published:** 2024-03-30

**Authors:** Quanle Zou, Weizhi Wang, Xin Wang

**Affiliations:** State Key Laboratory of Coal Mine Disaster Dynamics and Control, School of Resources and Safety Engineering, Chongqing University, Chongqing, 400044 China

**Keywords:** Cementing cement, Calcium lignosulfonate, Cement hydration, Modification mechanism, Solid Earth sciences, Civil engineering

## Abstract

During the construction of coalbed methane extraction wells, cementing cement sheath is crucial for the stability and sealing of surface wells. One effective method to enhance these properties is the addition of lignosulfonate. However, the mechanism of the effect of calcium lignosulfonate on the whole process of cement hydration is still unclear. In this paper, the water distribution and variation characteristics of calcium lignosulfonate modified cement paste were revealed by low-field nuclear magnetic resonance technology, and the hydration ion experiment of modified cement was carried out to obtain the variation characteristics of hydration ions of modified cementing cement. Finally, the formation mechanism of hydration products was clarified by analyzing the phase change of modified cement stone. The results indicate that the cement paste’s hydration process can be divided into four stages: dissolution, crystallization, acceleration, and decline. During the dissolution stage, calcium lignosulfonate’s air entraining effect maintains the cement paste in a stable suspension state. In the crystallization stage, calcium lignosulfonate’s electro-repulsion delays the formation of hydration products and the hydration process. During the acceleration stage, the addition of calcium lignosulfonate reduces bound water formation in the cement slurry’s flocculation structure, and the released filled water participates more in the hydration reaction, reducing the total relaxation signal’s increasing trend. In the decline stage, the cement paste has solidified, and the system’s water is primarily in the porous medium. The research results have practical guiding significance for the addition of calcium lignosulfonate in cementing operations.

## Introduction

As fossil energy resources deplete, the focus has shifted towards the construction of new energy sources. Coalbed methane, a clean and high-quality energy source with vast reserves, has significant development potential. Its industrial development is crucial for energy security, coal mine safety, and achieving the ‘double carbon’ goal. Exploiting coalbed methane not only enhances the utilization rate of coal resources but also reduces greenhouse gas emissions, improves mine safety, and promotes energy structure optimization^1^. Coalbed methane mining involves underground extraction and surface well extraction. The latter, characterized by ground construction, pre-mining completion, non-interference with coal production, high methane concentration, large production scale, and stable production, has seen rapid development globally. However, surface well extraction can disturb the overlying strata during the extraction process, and local stress field disruption can increase the surface well’s axial pressure. When the surface well is squeezed and destroyed, its extraction efficiency is significantly affected^2,3^. Therefore, cementing measures are necessary to enhance surface well stability and ensure efficient coalbed methane extraction.

Cementing, which involves injecting cement slurry into the annular space between the casing and the wellbore and allowing it to solidify into a cement sheath, is crucial for preventing casing deformation and external material immersion. The cement sheath’s performance, a significant aspect of cementing measures, influences the stability and sealing of surface wells. However, ordinary cement sheaths, being brittle materials, have low early strength and poor compactness^4,5^. To address these shortcomings, additives are typically incorporated into the cement slurry to enhance its overall performance^6^. The cement active additives can significantly improve the strength of cement stone. Common cement active additives include silica fume, fly ash, zeolite, lignosulfonate and so on. Xu, WB et al.^7^ studied the effect of silica fume on the mechanical properties and microstructure of cement stone. Eker, H et al.^8,9^ explored the weakening effect of fly ash fineness on the alkali-silica reaction (ASR) of concrete, and proved that the fineness of fly ash and the substitution rate in concrete can reduce the ASR. The influence mechanism of zeolite on the strength of cement stone was studied. Lignosulfonate is one of the earliest and most widely used cement dispersants. As a multi-component polymer anionic surfactant, calcium lignosulfonate has attracted much attention due to its strong dispersibility and cohesiveness^10–12^.

The hydration process of cement significantly influences the macroscopic properties of cement-based materials. During this process, the cement’s microstructure evolves, and its mechanical properties begin to form and develop^13^. Numerous studies have examined the impact of lignosulfonate on cement hydration^14–17^. For instance, Serina Ng et al.^18^ used a rheometer and isothermal calorimetry to study the effect of lignosulfonate on the early rheological and hydration properties of cement paste, quantitatively demonstrating lignosulfonate’s early hydration effect on cement. Similarly, Tobias Danner et al.^14^ used isothermal calorimetry, in-situ XRD, and thermal analysis to investigate the impact of calcium lignosulfonate on the phase change of ordinary Portland cement during early hydration. Mezhov A et al.^19^ studied the effect of sodium lignosulfonate on the early hydration of cement with two different C_3_A contents. Given the many components of cement clinker, the addition of calcium lignosulfonate complicates the hydration process. Therefore, real-time characterization of water migration and phase composition during different hydration reaction stages is crucial for revealing the hydration mechanism.

Nuclear magnetic resonance (NMR) technology, an efficient and continuous non-destructive test, has been extensively applied in the study of cement-based materials^20–22^. It has been used for characterizing cement gel growth and for using the evolution of the T_2_ signal to characterize different hydration stages^19,20,22,23^. Its cross-referencing with techniques such as X-ray diffraction can be used to reveal phase changes in the hydration process of cement-based materials^24^. Q. Liu et al.^25^ studied the effect of nano-silica on the hydration kinetics of CaO. Gastaldi and She et al.^26,27^ used X-ray diffraction and scanning electron microscopy to verify the accuracy of NMR testing on cement hydration characterization. Additionally, methods such as conductivity and pH testing can quantitatively analyze the cement hydration process, reflecting the change in ion concentration and hydration rate during cement hydration. Therefore, this study applies NMR technology to the investigation of cement hydration, combined with conductivity, pH, XRD, and other testing methods, to provide a more comprehensive understanding of the hydration mechanism of calcium lignosulfonate modified cement.

In summary, there are few studies on the hydration process of calcium lignosulfonate modified cement, and the hydration mechanism of calcium lignosulfonate modified cement is still unclear. In this study, nuclear magnetic resonance technology was utilized to uncover the water distribution and variation characteristics of a cement paste modified with calcium lignosulfonate. This was complemented with conductivity and pH value plasma test experiments to reveal the ion transformation behavior during the hydration process of the modified cement. Finally, the phase change characteristics of modified cement stone were analyzed by X-ray diffraction analysis, and the formation mechanism of modified cement hydration products was revealed. The research results can provide theoretical support for improving the performance of cementing cement stone and the efficient exploitation of coalbed methane.

## Materials and methods

### Cement and dispersants

Grade G high sulfate resistant cement was used in the experiment,, which can be mixed with additives and is suitable for most cementing operations. Its mineral composition primarily includes tricalcium aluminate (C_3_A), tricalcium silicate (C_3_S), dicalcium silicate (C_2_S), and tetracalcium aluminoferrate (C_4_AF). The content of C_3_S is 48%-65%, the content of C_3_A is < 3%, the content of C_4_AF + 2C_3_A is < 24%, and the content of alkali is < 0.75%. Cement strength is mainly determined by C_3_S. After the cement is injected into the well, the strength can be reached in a short time, and the optimized cement has good stability.

The dispersant used in this study is calcium lignosulfonate, a natural polymer extracted from waste paper pulp. Calcium lignosulfonate exhibits strong dispersion effects on cement paste and also has retarding properties. This makes it an effective additive for enhancing the properties of cement paste.

### Testing principle and method

#### T_2_ value measurement

Nuclear magnetic resonance (NMR) tests typically employ T_1_ transverse relaxation time and T_2_ transverse relaxation time. Compared with T_1_ relaxation time, the detection speed of T_2_ relaxation time is faster, and the test results of the two are basically the same. Therefore, the current nuclear magnetic resonance test is dominated by T_2_ relaxation time. According to the NMR relaxation mechanism, the transverse relaxation time T_2_ value primarily comprises several aspects, as shown in Formula (1).

1 $$\frac{1}{{T}_{2}}=\frac{1}{{T}_{2B}}+\frac{1}{{T}_{2S}}+\frac{1}{{T}_{2D}}$$

In the formula, T_2B_ is the volume ( free ) relaxation time, ms ; T_2S_ is the surface relaxation time, ms ; T_2D_ is diffusion relaxation time, ms.

The value of T_2B_ is usually 2 ~ 3s, which is much larger than T_2_, so the first term on the right side of formula (1) can be ignored. When the magnetic field is very uniform, T_2D_ can also be ignored. Therefore, T_2_ is equivalent to T_2S_, so Eq. (1) can be simplified as Eq. (2)^28^ :

2 $$\frac{1}{{T}_{2}}={\rho }_{2}\left[\frac{S}{V}\right]$$

In the formula, S is the surface area of the pore, μm^2^ ; V is the volume of the pore, μm^3^ ; ρ_2_ is the transverse surface relaxation strength of cement, μm / ms.

#### Determination of conductivity and pH value

The conductivity, a physical quantity that characterizes charge mobility in a solution, is measured in S/m. It is proportional to the ion concentration of the solution. The components in the cement undergo a hydration reaction with the addition of water, resulting in hydration ions, which can lead to changes in the conductivity of the cement. A conductivity meter can measure the ion concentration of the solution. The measurement principle of the conductivity meter involves placing two parallel plates into the solution to be measured, applying a certain potential at both ends of the plate, and measuring the current flowing between the plates^29^. According to Ohm’s law, the conductivity (G) is the reciprocal of the resistance (R) and is determined by the voltage and current.

The pH value is a crucial indicator of the hydration reaction in cement. The Ca(OH)_2_ and other ions generated in the hydration reaction of cement will affect the stability of cement slurry. The change of pH value will also affect the viscosity, fluidity and other properties of cement slurry. Therefore, maintaining an appropriate pH value is one of the important factors to ensure the stability of cement slurry. In this paper, the pH value of cement slurry is measured by pH measuring instrument. The measuring strip is placed in the cement paste, and then the current pH value is indicated^30,31^.

#### X-ray diffraction analysis

X-ray diffraction analysis (XRD) is a method used to characterize crystal structures and their changes. It is applied in various fields such as materials science, chemistry, biology, medicine, ceramics, metallurgy, and mineralogy. When a monochromatic X-ray beam is incident on a crystal, it interacts with the crystal’s atoms, which are arranged in regular cells. The distance between these atoms is comparable to the wavelength of the incident X-ray. As a result, X-rays scattered by different atoms interfere with each other, producing strong X-ray diffraction in specific directions. The orientation and intensity of the spatial distribution of these diffraction lines are closely related to the crystal structure. Each crystal produces a unique diffraction pattern that reflects the atomic distribution within the crystal^32,33^. This is the fundamental principle of X-ray diffraction. By comparing the XRD pattern with a standard pattern, the phase composition of cement can be determined.

### Methods of experiment

G-grade cement of the same quality was selected, and calcium lignosulfonate with mass fractions of 0%, 0.1%, 0.2%, 0.3%, and 0.4% was added respectively^34^. According to the ratio of water-cement ratio of 0.44, water and the above cement and calcium lignosulfonate were uniformly mixed through a stirrer to prepare five cement slurry samples^35,36^. The cement slurry numbers were 0 #, 1 #, 2 #, 3 #, and 4 #.

**Figure 1:**
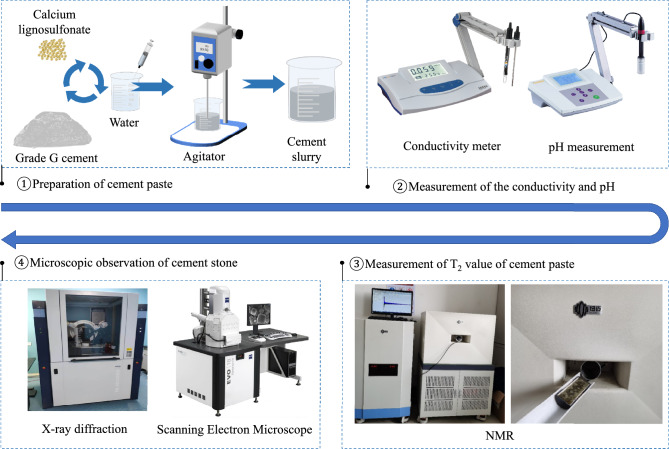
Experimental process.

As shown in Fig. 1, the effects of different mass fractions of calcium lignosulfonate on the conductivity and pH value of cement slurry were determined by conductivity meter and pH meter. For conductivity measurements, each cement slurry sample was poured into a mud cup. The conductivity meter electrode was cleaned and then inserted into the cement slurry sample. After gentle stirring and allowing the sample to settle, the reading on the display screen was taken as the conductivity of the cement slurry once the displayed value stabilized. For pH measurements, the pH meter probe was cleaned and calibrated using different standard solutions. The probe was then cleaned again, dried with filter paper, and inserted into the cement slurry sample. The subsequent reading was taken as the pH value of the cement slurry.

The experiment utilized a Newman company’s NMR nuclear magnetic resonance analyzer. The test was performed according to GB / T 42035–2022. The permanent magnet had a magnetic induction intensity (B) of 0.53 T, a proton resonance frequency of 23 MHz, and a constant temperature control of 32 °C. The echo number of the CPMG pulse sequence was n = 12000, and the half echo time was τ = 110 μs, which was used to record a series of echo sequence times. Each CPMG signal was scanned 32 times on average. The repeated sampling waiting time (TR) was 1500 ms. The collected relaxation data were processed using inversion software to obtain the T_2_ value distribution curve of the sample. Simultaneously, the T_2_ value of the whole hydration process of each cement paste was tested by NMR using CPMG sequence.

After curing for 24 h, the solidified cement stone is cut into blocks with dimensions ranging from 1cm x 1 cm to 2 cm x 2 cm and a thickness of less than 1cm. The sample table and cover glass are cleaned with alcohol cotton and allowed to dry naturally. The sample is placed in the center of the sample table’s groove, ensuring that the height of the sample is level with the sample table, and then it is pressed with the cover glass. The XRD instrument door is opened, the sample table with the sample is placed in the groove, and the door is locked to perform the XRD test. Once the test is completed, the sample is removed and the instrument is shut down. The phase composition of the cement samples can be determined through the XRD test.

## Results and discussion

### Cement paste T_2_ distribution curve

**Figure 2:**
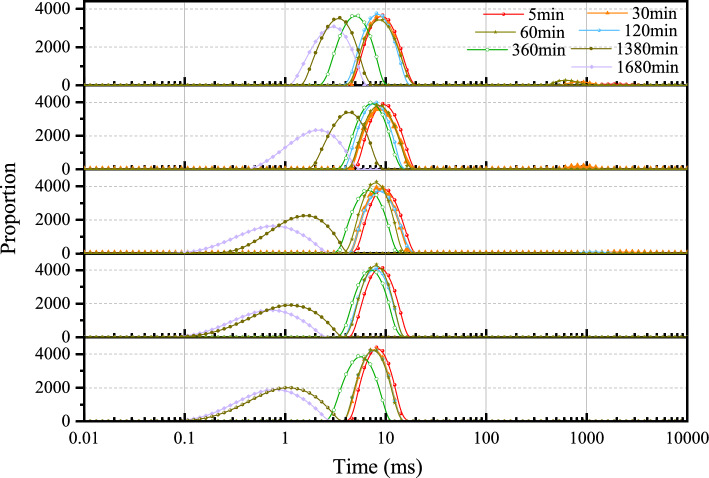
T_2_ value distribution of cement paste in different periods.

The T_2_ values of five types of cement pastes (0 #, 1#, 2#, 3#, 4#) during the hydration process were monitored using NMR, with the results displayed in Fig. 2. The T_2_ spectrum primarily consists of two relaxation peaks in the early stages of hydration. The first relaxation peak ranges from 0.5 to 10 ms, and this range is defined as the first relaxation peak. The second relaxation peak occurs within the 1000–5000 ms interval, and the peak within this range is defined as the second relaxation peak.

The T_2_ value of the first relaxation peak at the initial stage of hydration reaction for all cement pastes is approximately 10 ms. As the hydration reaction progresses, the peak value shifts towards the short relaxation direction, and this shift decreases with the addition of calcium lignosulfonate. In the 0# sample, when the hydration time reaches 1680 min, the peak of the first relaxation peak appears near 0.76 ms. In the 3# sample, at the same hydration time, the peak of the first relaxation peak is near 2.31 ms. In the 4 # sample, when the hydration reaction was carried out to 1680 min, the peak position of the first relaxation peak was near 4.64 ms. Moreover, the distribution of the first relaxation peak of each hydration time period in the 4# sample is more concentrated. For the second relaxation peak, it is not prominent in the 0# and 1# samples. With the addition of calcium lignosulfonate, the second relaxation peak gradually appears in the 3# sample, and its area increases with the increase of the mass fraction of calcium lignosulfonate.

### Relaxation signal variation characteristics

**Figure 3:**
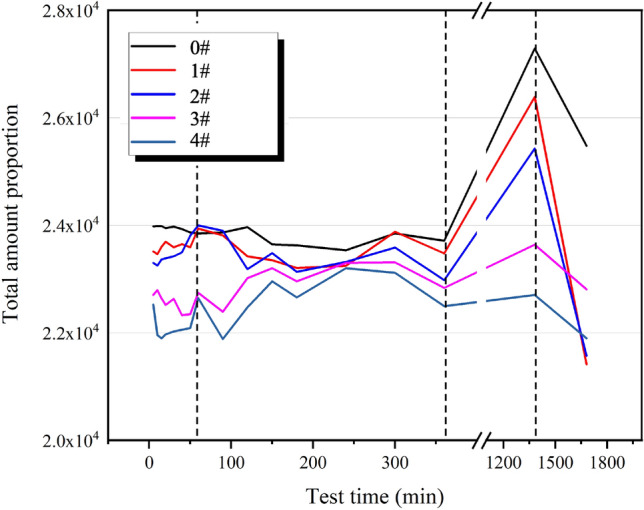
Time-varying law of total relaxation signal.

The total T_2_ measured throughout the hydration process of each cement paste was analyzed to characterize the overall relaxation characteristics of the cement paste. As depicted in Fig. 3, the T_2_ value exhibited a gradual decrease with the addition of calcium lignosulfonate during the hydration reaction. In the 0–30 min of the hydration reaction, the total T_2_ signal of each cement paste went through a fluctuation stage. During the 30–360 min of the hydration reaction, the total T_2_ signal of each cement paste entered a fluctuating upward stage. In the 360–1380 min of the hydration reaction, the total T_2_ signal of each cement paste increased most rapidly, and when the hydration reaction reached 1380 min, the total T_2_ signal of each cement paste reached the maximum value of the entire hydration reaction. After 1380 min of the hydration reaction, the total T_2_ signal of each cement paste decreased rapidly.

### Moisture variation characteristics of hydration process

The hydration process of cement slurry involves changes in the confined state of water. The time (relaxation time) it takes for ^1^H in water molecules to transition from a high-energy state to an equilibrium state after external energy excitation also changes with the confined state. Therefore, the relaxation time can somewhat reflect the characteristics of water state changes during the cement hydration process.

In Fig. 2, the first relaxation peak in the relaxation time range of 0.1–5 ms is generated by the bound water of the flocculated structure in the cement paste. The second relaxation peak, with a relaxation time range of 5–15 ms, is generated by the water filled between the cement flocculated structures. Finally, the third relaxation peak, with a peak at about 1000 ms, represents free water.

In Fig. 2, with the increase of hydration time, the first relaxation peak moves to the direction of short relaxation time, indicating that the bound water between the flocculation structures of cement slurry is reduced, water molecules enter the gel structure, and the constraint of water molecules is enhanced, thus shortening the relaxation time. In the whole early hydration process, with the addition of calcium lignosulfonate, the trend of the first relaxation peak moving to the short relaxation direction gradually decreases, and the distribution of the first relaxation peak of the 4 # sample has been concentrated near 10ms, indicating that the addition of calcium lignosulfonate increases the proportion of flocculation structure filling water in cement slurry. In addition, the addition of calcium lignosulfonate reduces the area of the first relaxation peak, and it can be observed that the second relaxation peak near 1000 ms is increasing, which indicates that the addition of calcium lignosulfonate can promote the dispersion of cement particles and the destruction of flocculation structure. The water molecules are released, which increases the content of free water in the cement slurry system.

**Figure 4:**
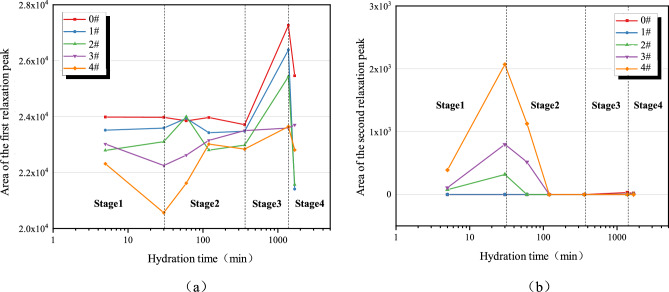
Relaxation peak area variation characteristics: (**a**) The first relaxation peak; (**b**) The second relaxation peak.

The variation characteristics of the peak areas of the first and second relaxation peaks of the cement sample are further compared and analyzed, as shown in Fig. 4. The peak area of the first relaxation peak initially decreases and then increases, while the peak area of the second relaxation peak initially increases and then decreases until it disappears. The decrease and increase were significantly greater for the cement paste with calcium lignosulfonate added to it. This indicates that as the mass fraction of calcium lignosulfonate increases, the changes in the two types of water in the cement paste become more severe, accelerating the formation of free water in the cement paste. With the addition of calcium lignosulfonate, it will coat the surface of cement particles, hindering the dissolution of clinker in the particles, thus delaying the formation of hydration products. Therefore, in the 0–30 min stage, the peak area of the first relaxation peak gradually decreases and the peak area of the second relaxation peak gradually increases. In the hydration reaction of 30–360 min, the hydration products begin to form. Free water in the cement slurry system participates in the hydration reaction, and water molecules enter the flocculation structure. Therefore, the area of the second relaxation peak gradually decreases, and the area of the first relaxation peak rebounds.

### Modified cementing cement hydration ion change characteristics and phase change

**Figure 5:**
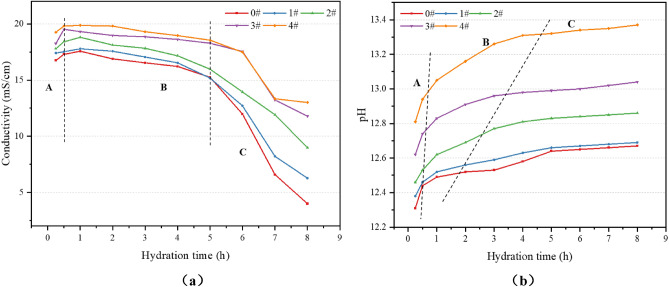
Effect of calcium lignosulfonate on the electrical conductivity and pH value of cement paste : (**a**) Electrical conductivity ; (**b**) pH value.

When cement comes into contact with water, its components begin to dissolve. At this point, the liquid phase filled between the particles is no longer pure water, but a solution containing ions such as Ca^2+^, OH^−^, SO_4_^2−^, and Al(OH)_4_^−^. The conductivity of cement represents the concentration and movement speed of conductive ions in the cement. The change in the pH value of the cement paste reflects the change in the cement hydration rate. By testing the conductivity and pH value of five cement pastes of 0 #, 1 #, 2 #, 3 # and 4 #, the effect of calcium lignosulfonate on the conductivity and pH value of cement is shown in Fig. 5.

As seen in Fig. 5(a), during stage A, the concentration of Ca^2+^, OH^-^, SO_4_^2−^, Al(OH)_4_^−^ in the cement paste increases as time progresses after the cement and water are mixed, resulting in an increase in electrical conductivity. The addition of calcium lignosulfonate causes the conductivity curve to rise. This is because calcium lignosulfonate ions are adsorbed onto the surface of cement particles, gradually encapsulating them, which delays cement hydration and results in an increase in the conductivity of the cement paste. At the end of stage A, there’s a sudden turning point (the highest point), marking the transition into stage B. In stage B, the electrical conductivity of the cement slurry shows a slow downward trend, indicating that ettringite and Ca(OH)_2_ begin to crystallize, the ions in the solution start to decrease, and the liquid phase space also begins to decrease. The ion release rate during the hydration process of cement clinker minerals is slower than the cation consumption rate during the crystallization process. In stage C, the electrical conductivity of the cement slurry decreases rapidly, indicating that crystals such as ettringite and Ca(OH)_2_ begin to form in large quantities as the hydration reaction proceeds. The addition of calcium lignosulfonate reduces the rate of decrease in cement slurry conductivity. This is because the calcium lignosulfonate molecule, being a polymer molecule, adsorbs onto the surface of the cement particles, hinders the growth of the hydration products, delays the hydration process, and thus reduces the rate of decrease in the conductivity of the cement slurry. Therefore, the addition of calcium lignosulfonate changes the accumulation state and internal structure of cement particles, thereby affecting the conductivity.

As depicted in Fig. 5(b), the variation characteristics of the cement slurry pH can also be divided into three stages. In stage A, with the mixing of cement and water, the dissolution of Ca^2+^, OH^-^, SO_4_^2−^, Al(OH)_4_^−^ ions in the cement paste leads to a rapid increase in the pH value. In stage B, the pH of the cement paste continues to rise, but at a reduced rate, indicating that OH^-^ has been gradually transformed into Ca(OH)_2_ crystals during this stage. After the end of stage B, the pH of the cement slurry tends to stabilize, marking the transition into stage C. In stage C, the pH value changes more smoothly, as most of the OH^-^ ions in the cement paste have formed Ca(OH)_2_ crystals. The addition of calcium lignosulfonate significantly increases the pH value of the cement slurry, indicating that calcium lignosulfonate can stabilize a large number of Ca^2+^ and OH^−^ ions in the solution. Simultaneously, the addition of calcium lignosulfonate delays the time of pH entering stage C. This delay is due to the fact that Ca(OH)_2_ in the liquid phase can only crystallize at extremely high supersaturation during the hydration of C_3_S. The addition of calcium lignosulfonate causes the silicate anion and calcium lignosulfonate anion (SO_4_^2−^) to affect the nucleation process of Ca(OH)_2_, hindering the nucleation and development of Ca(OH)_2_. The Ca(OH)_2_ supersaturated solution can remain stable, and there are a large number of stable Ca^2+^ and OH^-^ ions in the solution, which increases the system’s pH value.

**Figure 6:**
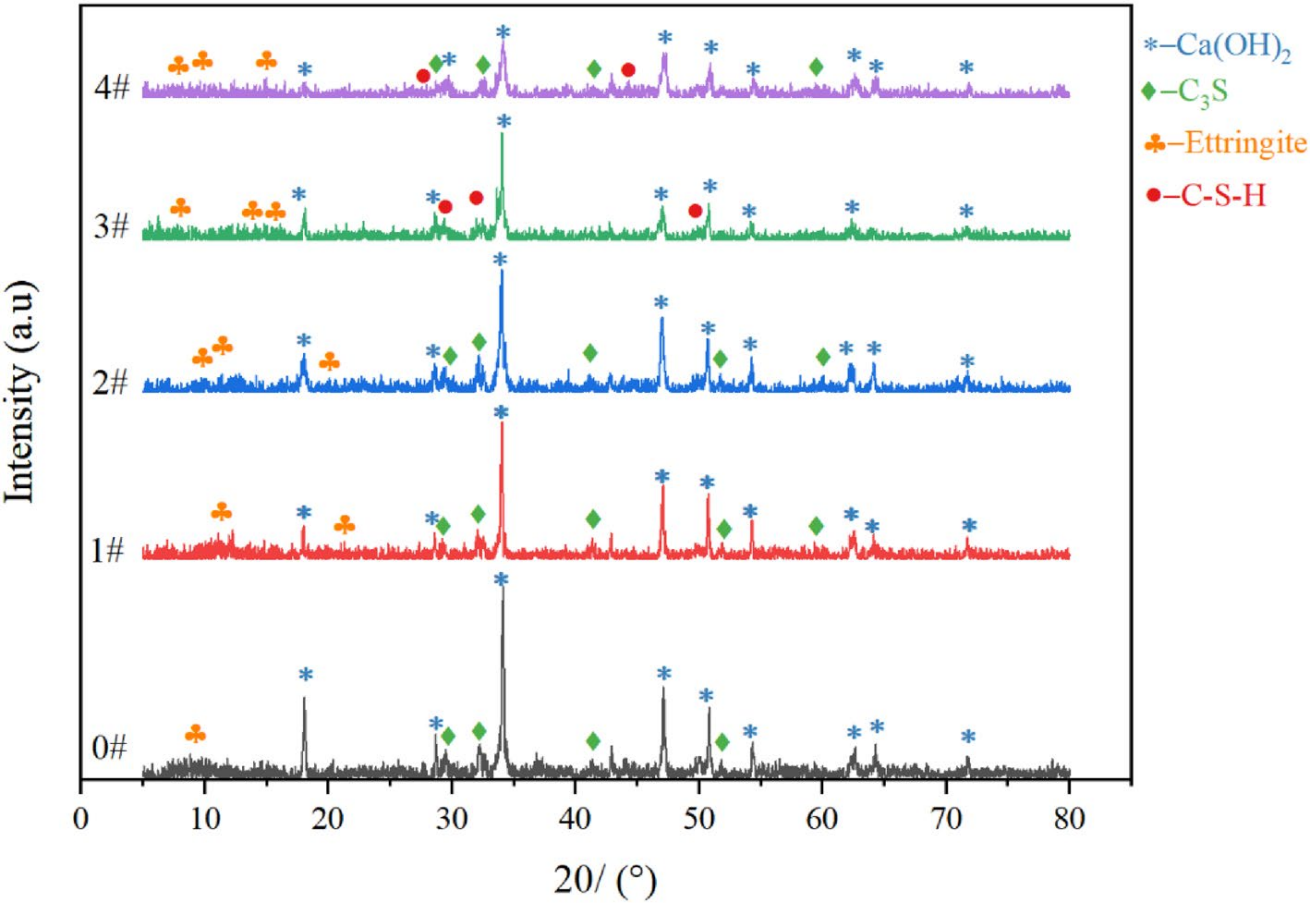
XRD pattern of cement sample.

The hydration reaction of cement commences upon contact with water. The cement components (C_3_A, C_3_S, C_2_S, C_4_AF) and calcium sulfate gradually hydrate to form Ca(OH)_2_, C-S-H, and ettringite. Figure 6 presents the XRD pattern of cement samples hydrated for 24 h under curing conditions. In the absence of calcium lignosulfonate, the hydration products are primarily Ca(OH)_2_ and a small amount of ettringite and unhydrated C3S. With the addition of calcium lignosulfonate, the diffraction peak intensity of Ca(OH)_2_ in cement samples gradually decreases. Simultaneously, the diffraction peak intensity of C_3_S significantly increases, and the diffraction peak intensity of ettringite also shows an increasing trend. C-S-H diffraction peaks appear in 3# and 4# cement samples. According to the test results and the research results of Yang and Colombo et al., calcium lignosulfonate can promote the hydration of C_3_A, and the generated ettringite precipitates on the surface of cement particles, thus hindering the hydration process of C_3_S and reducing the production of Ca(OH)_2_.

### Modification mechanism of calcium lignosulfonate on cement hydration

**Figure 7:**
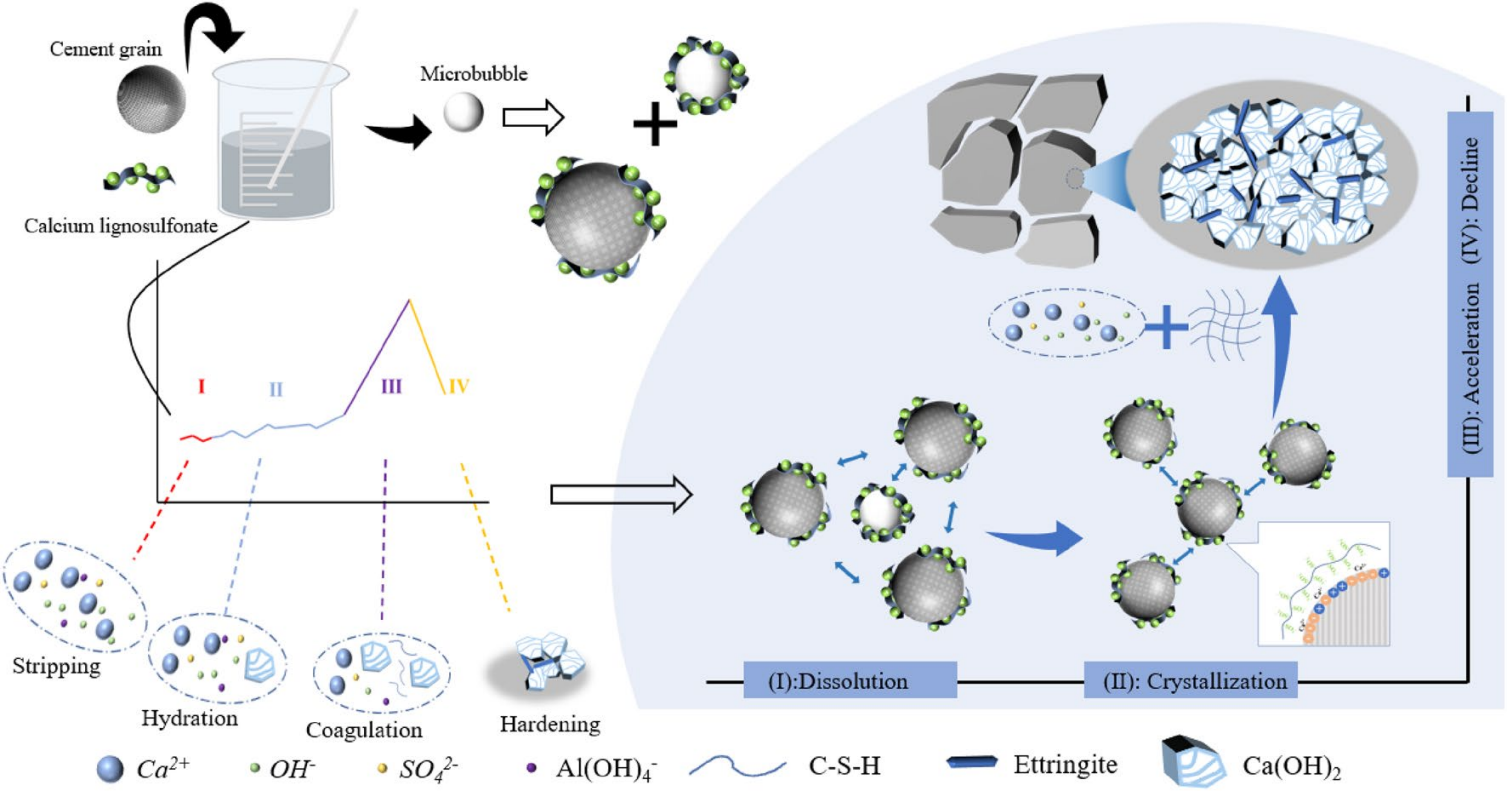
Four-stage hydration characteristics of modified cement paste.

As depicted in Fig. 7, the hydration process of cement slurry can be divided into four specific reaction stages based on the change law of the relaxation signal of the cement slurry and the change characteristics of hydration ions and phase changes of calcium lignosulfonate modified cementing cement. These stages are: I dissolution stage, II crystallization stage, III acceleration stage, and IV decline stage.

(1) The dissolution stage (I): 0–30 min.

**Figure 8:**
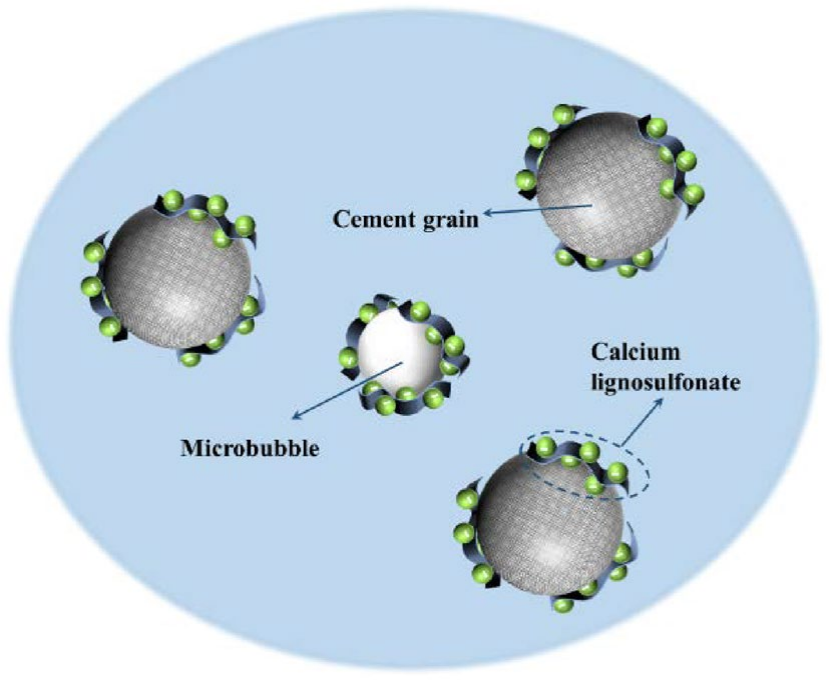
Lubrication effect caused by polar air bubbles.

During this stage, the total signal of cement slurry relaxation exhibits minor fluctuations, and with the addition of calcium lignosulfonate, the total signal of cement slurry relaxation shows a decreasing trend. This stage corresponds to stage A in Fig. 5. In this stage, the early hydration reaction of the cement slurry is low. After the cement is mixed with water, the cement slurry forms a flocculation structure, encapsulating a portion of the free water within the cement particles. At this stage, the Ca^2+^, OH^-^ plasma in the cement slurry begins to appear. Additionally, calcium lignosulfonate, an anionic surfactant, decomposes into negatively charged anions in the alkaline medium of the cement slurry. These anions will be adsorbed by cement particles to form a layer of dissolved film, and the air entraining effect of calcium lignosulfonate will introduce a certain amount of bubbles during the stirring process. As shown in Fig. 8, these microbubbles are surrounded by a molecular film of the dispersant and have the same charge symbol as the cement particles. The electric repulsion force between the bubble and the cement particles disperses the cement particles. Under the action of the electric repulsion force, the cement slurry will be in a relatively stable suspension state, destroy the flocculation structure of the cement slurry and release the water in it. Therefore, at the end of this stage, the second relaxation peak of the cement slurry reaches the maximum value, and the free water content in the cement slurry system increases.

(2) The crystallization stage (II): 30-360 min.

**Figure 9:**
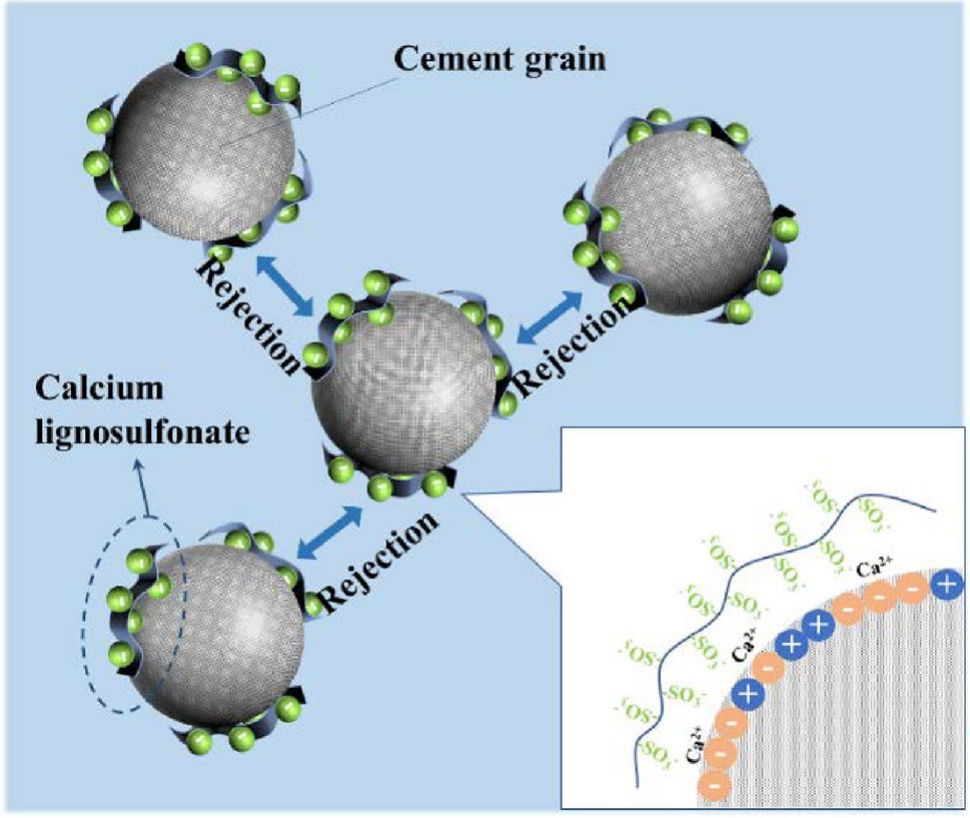
Schematic diagram of the adsorption mechanism of calcium lignosulfonate on cement particles.

At this stage, the total signal of cement slurry relaxation exhibits a fluctuating state. However, with the addition of calcium lignosulfonate, the total signal displays a gradually decreasing trend. This stage corresponds to the B stage in Fig. 5. During this stage, the concentration of Ca^2+^ and OH^-^ ions in the cement paste continuously increases, reaching a supersaturated state. Consequently, ettringite and Ca(OH)_2_ crystals begin to crystallize. The initially formed hydration products envelop the surface of the cement particles, impeding the dissolution of the cement particles and thereby delaying ion formation. At this stage, the number of hydration products is not substantial, resulting in a more gentle change in the total relaxation signal of the 0 # sample. The sulfonic acid group of calcium lignosulfonate acts as a complex formation group, forming a more stable complex with Ca^2+^ in an alkaline medium, as depicted in Fig. 9. This complex increases the diffusion resistance of water molecules, leading to an increase in the diffusion barrier and a decrease in the diffusion coefficient. The hydrophobic layer adsorbed on the surface of cement particles also reduces the likelihood of collisions between liquid water molecules and unhydrated cement particles, resulting in a slower cement dissolution rate and a lower hydration rate. Therefore, the growth of hydration products and specific surface area in the slurry is minimal, which is the primary reason why the total amount of T_2_ in the cement sample in Fig. 3 changes more gently within 360 min. Simultaneously, the dispersant particles adsorbed on the surface of the cement particles carry the same charge, leading to the dispersion of the cement particles due to the electrical repulsion between them.

(3) The acceleration stage (III): 360–1380 min.

At this stage, the total signal of cement slurry relaxation rapidly increases. However, with the addition of calcium lignosulfonate, this growth trend diminishes. During this stage, most of the ions in the cement slurry have formed hydration products such as Ca(OH)_2_ crystals and ettringite. At this point, the water filled in the flocculation structure of the cement slurry has been completely released and participates in the hydration reaction with the free water in the system to form bound water. Consequently, the total signal of cement slurry relaxation surges. Correspondingly, the conductivity of the cement slurry rapidly decreases. The addition of calcium lignosulfonate impedes the growth of hydration products due to its adsorption on the surface of cement particles, delays the hydration process, reduces the formation of bound water in the flocculation structure of the cement slurry, and releases the filled water. This results in more involvement in the hydration reaction, thereby reducing the trend of increasing the total relaxation signal of the cement slurry. At this stage, the mechanical structure of the cement sample is gradually formed.

**Figure 10:**
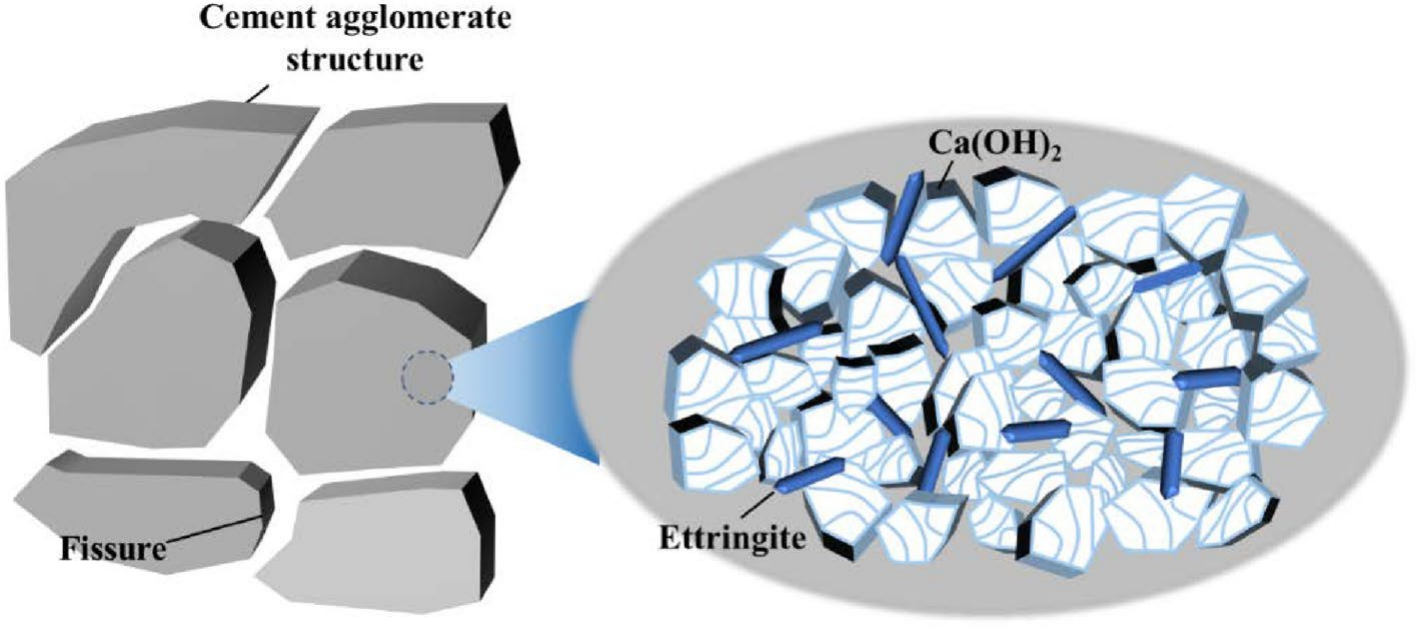
Ettringite and Ca(OH)_2_ on the surface of cement particles.

Further analysis of the hydration ion characteristics of modified cement and the phase change characteristics of modified cement stone reveals that when calcium lignosulfonate is added to the cement system, the molecules of calcium lignosulfonate are primarily adsorbed on the surface of gypsum and aluminate. This hinders the transformation of hemihydrate gypsum and anhydrous gypsum into dihydrate gypsum. Due to the higher solubility of hemihydrate gypsum and anhydrous gypsum in the cement slurry system, more Ca^2+^ and SO_4_^2−^ dissolve in the cement system, promoting the formation of ettringite and its precipitation on the surface of cement particles. Simultaneously, the sulfonic acid group in calcium lignosulfonate competes with SO_4_^2−^ in the reaction with C_3_A, thus promoting the formation of ettringite. As depicted in Fig. 10, the addition of an appropriate amount of calcium lignosulfonate promotes the mutual attraction of C-S-H gel in cement stone to form a space grid and encourages the formation of a large amount of ettringite. When the mass fraction of calcium lignosulfonate exceeds 0.3%, the C-S-H gel becomes flocculent^37^. This indicates that an excessive amount of calcium lignosulfonate will inhibit the crystal and structure growth of hydration products, especially making it difficult for C-S–H gel to form a connected space grid. The retarding effect of a high mass fraction of calcium lignosulfonate on the early hydration of cement inhibits the early formation of C-S-H gel and Ca(OH)_2_ crystal. At the same time, Ca(OH)_2_ crystals and C-S-H gels agglomerate together, generating more pore structures between the agglomerated structures. This is because the air-entraining effect of calcium lignosulfonate can reduce the gas–liquid interfacial tension. Therefore, the leading role of different mass fractions of calcium lignosulfonate varies.

(4) The decline stage (IV): 1380–1680 min.

At this stage, the total signal of cement slurry relaxation exhibits a trend of rapid attenuation. At this point, the filling water released by the flocculation structure of the cement slurry is continuously consumed to generate hydration products, indicating that the cement slurry has condensed into a consolidated body at this stage. Apart from a small amount of chemically bound water, the system mainly consists of water present in the porous medium.

In summary, the addition of an appropriate amount of calcium lignosulfonate promotes the generation of hydration products such as Ca(OH)_2_ crystals and ettringite crystals. A large number of interlaced, needle-like ettringite crystals are formed, with Ca(OH)_2_ crystals embedded within them and filling the cracks between the cement agglomerates. This significantly improves the compactness of the cement matrix material. However, with the addition of excessive calcium lignosulfonate, the air entraining effect and electrical repulsion of calcium lignosulfonate take precedence in the cement hydration reaction. This makes it difficult for C-S–H gel to form a connected space grid, resulting in a large number of pores between cement particles.

## Conclusions

In this paper, the effect of calcium lignosulfonate on the hydration reaction process of cement was clarified, and the ion transformation behavior in the hydration process of modified cement was revealed. The formation mechanism of hydration products was clarified by analyzing the phase change of modified cement stone. The research results are of great significance to the study of the nucleation mechanism of calcium lignosulfonate modified cement hydration products. The main conclusions are as follows :

- (1) The addition of calcium lignosulfonate can promote the increase of free water content in the hydration process. The decrease of the total amount of T_2_ relaxation signal with the addition of calcium lignosulfonate reflects the process of the transformation of physically bound water into chemically bound water in the cement slurry system.
- (2) The adsorption of calcium lignosulfonate on the surface of cement particles delays the hydration process of cement slurry, resulting in a decrease in the rate of decrease in the conductivity of cement slurry. At the same time, the poisoning effect of calcium lignosulfonate anion (SO_4_^2−^) on the nucleation process of Ca(OH)_2_ hinders the crystallization of Ca(OH)_2_, which in turn increases the pH value of the system.
- (3) The different mass fractions of calcium lignosulfonate have different leading effects on cement. The addition of appropriate amount of calcium lignosulfonate will promote the mutual attraction of C-S–H gel in cement stone, promote the formation of ettringite in large quantities, and precipitate on the surface of cement particles. The addition of excessive calcium lignosulfonate will make the air-entraining effect and electrical repulsion of calcium lignosulfonate play a leading role in the cement hydration reaction.
- (4) By analyzing the total relaxation signal of T_2_, the definition and time distribution of the four stages of calcium lignosulfonate modified cement hydration were proposed : dissolution stage (0–30 min), crystallization stage (30–360 min), acceleration stage (360–1380 min) and decline stage (1380–1680 min). The leading role of each stage in the whole process of modified cement hydration was clarified.

Although the hydration mechanism of calcium lignosulfonate was studied from the hydration process and hydration products, hydration is an exothermic reaction. Therefore, it is strongly suggested that further research should be carried out on the nucleation and growth process of hydration products from the perspective of hydration kinetics.

## Data Availability

The datasets used and/or analysed during the current study available from the corresponding author on reasonable request.
